# Assessing the dimensions of psychological (in)flexibility in adolescence: Validation of the Portuguese Version of the Multidimensional Psychological Flexibility Inventory - short form

**DOI:** 10.1192/j.eurpsy.2024.676

**Published:** 2024-08-27

**Authors:** M. Cunha, S. Flórido, C. Pinto-Gouveia, A. Galhardo

**Affiliations:** ^1^ISMT; ^2^CINEICC, UC; ^3^Clinical Psychology, ISMT; ^4^Psychiatry Department, UC, Coimbra, Portugal

## Abstract

**Introduction:**

Psychological Flexibility (PF) is a complex and extensively studied concept within the Acceptance and Commitment Therapy (ACT) framework. PF denotes one’s capacity to effectively navigate psychological distress and challenges while aligning one’s actions with deeply held values. Given its association with mental health and overall well-being, it is crucial to develop assessment tools able to capture the various facets of flexible behaviour and design strategies for its enhancement.

**Objectives:**

To adapt the Portuguese version of the Multidimensional Psychological Flexibility Inventory (MPFI-24; Grégoire et al., 2020) for the adolescent population.

**Methods:**

The study involved 269 adolescents aged 12 to 18 years old. Participants completed a set of self-report instruments, including the MPFI24-A, the Depression, Anxiety, and Stress Scales-21 (DASS-21), Mental Health Continuum - Short Form (MHC-SF), and the PsyFlex-A, which also assesses PF. A subsample also completed the MPFI24-A four weeks later to assess test-retest reliability.

**Results:**

Two models, specifically the six-factor correlated model and the bifactor model, emerged as presenting the best fit when analysing data separately for the Flexibility and Inflexibility indices. The MPFI24-A demonstrated good reliability for both overall scores (α = .90 and α = .85, respectively) and good test-retest reliability. The PF index showed significant positive associations with PsyFlex-A scores, perceived mental health, and a moderate negative association with depression and anxiety. Conversely, the Psychological Inflexibility (PI) index presented the opposite association pattern with these variables and showed no significant correlation with PF as measured by the PsyFlex-A. The two indices of the MPFI24-A demonstrated a weak positive correlation. Significant differences between boys and girls were found for the PF index, with boys showing higher scores. No significant differences were found between boys and girls concerning the PI index.

**Conclusions:**

Results suggest that the MPFI24-A is a reliable and valid instrument for assessing adolescents’ psychological flexibility and inflexibility competencies. Although further clarification of the MPFI24-A factor structure and the utility of different factors is warranted, the findings support its overall applicability.

**Disclosure of Interest:**

None Declared

